# An empirical analysis of the impact of early educational interventions before age three on cognitive development

**DOI:** 10.3389/fpsyg.2026.1629786

**Published:** 2026-05-13

**Authors:** Kexuan Yi, Wen Xiong, Jia-Jia Ou

**Affiliations:** School of Economics, Beijing Technology and Business University, Beijing, China

**Keywords:** children’s development, cognitive abilities, early educational interventions, mechanism test, rural households

## Abstract

Cognitive abilities are recognized as crucial for children’s learning and future development. This study investigates the impact of early educational intervention before age three on cognitive development among elementary school students. The sample data comprises students in grades 4–6, with an average age of 11 years, from 36 urban elementary schools and 9 township elementary schools in Hefei City, Anhui Province, China. The results show a significant “U-shaped” relationship between early educational interventions and children’s cognitive abilities, but this effect varies geographically. Early educational interventions before age three have a powerful effect on the cognitive abilities of children from non-rural households. In contrast, the effect on children from rural households is more limited. At the same time, this effect varies across income households. In addition, based on the school entrance test scores of these students when they entered elementary school, this study also explores the multiple mechanisms of action by which early educational interventions affect children’s cognitive abilities through influencing children’s pre-school reading ability (R) as well as children’s ability to write Chinese characters before school entry (WW). The findings suggest that China needs to provide differentiated instructional support for different groups of children to promote children’s cognitive development.

## 1 Introduction

Cognitive ability plays a crucial role in the key stages of early childhood development. International research on the cognitive ability of elementary school students emphasizes the application of diversification and standardized scales, and its measures mainly include situational memory ability (de Paula et al., 2015; Tomadesso et al., 2022), executive function (Ottonello et al., 2021; Dermody et al., 2020), and visual spatial skills (Ottonello et al., 2021; Muela et al., 2017). In contrast, Chinese elementary school students’ cognitive ability focuses on cultural adaptation and educational practices, which is measured by Chinese language learning tests (Chang, 2020), intelligence tests (Ma et al., 2022), and math scores (Lu et al., 2023; Wang et al., 2021). Summarizing the indicators of the above studies, we suppose that cognitive ability should include the following core dimensions: reading comprehension (Wang and Zheng, 2022), logical analysis (Wang and Zheng, 2022), memory (Sheridan et al., 2022), and language (Muela et al., 2017). Excellent cognitive skills help children acquire and process information more efficiently, which not only improve their reading and comprehension skills but also provide a strong foundation for their future learning journey (Caputi et al., 2016; Nemmi et al., 2016). At the same time, the cognitive abilities that children develop throughout their early years significantly influence their subsequent patterns of thinking. Therefore, scientifically guiding children’s cognitive development is a crucial strategy to set them on the road to success.

The development of children’s cognitive abilities is a complex process shaped by both internal and external factors. Children’s physiological maturity and genetic factors constitute the internal factors. Brain development directly influences children’s attention, memory, and thinking abilities (Goswami, 2008), which in turn affect cognitive development. Genetics can influence children’s intelligence and their cognitive abilities (Plomin and Petrill, 1997). However, considering internal factors alone is insufficient, the external environment is also critical. Research indicates that parents’ educational background, family economic status, and family atmosphere significantly promote children’s cognitive development (Bradley and Corwyn, 2002). Early literacy activities, book resources, and parental attitudes toward reading within the home literacy environment (HLE) can significantly enhance children’s reading interest and ability (Lee and Yeo, 2014). Furthermore, the frequency and quality of parent–child interactions also play a significant role in promoting language and cognitive skills (Price and Kalil, 2019). At the same time, socioeconomic status moderates the level of cognitive stimulation through resource accessibility. Lower socioeconomic status may limit access to educational and community resources, leading to insufficient cognitive stimulation (Lyon et al., 2003). More importantly, environmental factors not only independently influence children’s cognitive development but also exert significant moderating and constraining effects on the realization of genetic potential. Specifically, in supportive environments, genetic advantages are fully realized, but in deprived environments, cognitive development may be severely inhibited even in the presence of superior genetic endowment (Allegrini et al., 2022).

Among these external factors, early educational interventions play a crucial role. Early educational interventions help children overcome developmental obstacles and unlock their developmental potential through systematic measures. Early educational interventions have a significant promotional effect on children’s cognitive development, and the earlier they are implemented, the better the outcomes (Ou, 2005). Research indicates that early educational interventions before age three have both short-term and long-term impacts on children’s school preparation, academic performance, and social skills (Currie and Almond, 2011; Ajayi et al., 2017; Barnett, 2011). High-quality structured curricula can systematically enhance children’s mathematical, language, and social skills (Rege et al., 2018). At the same time, teacher quality, class size, and student-teacher ratios are key factors influencing the quality of early educational interventions and have a significant impact on the development of children’s executive functions and metacognitive abilities (Jiao et al., 2023). Although early educational interventions are theoretically significant, empirical research findings regarding their impact on cognitive abilities remain inconsistent (Mungas et al., 2018). On the one hand, some studies suggest that early educational interventions have significant long-term effects on children’s cognitive development. On the other hand, some studies suggest that early educational interventions have no significant impact on children’s cognitive abilities. Additionally, this discrepancy may be due to the heterogeneity of the research contexts. Much existing literature is based on contexts in developed countries, which are characterized by abundant educational resources and stable institutional environments. However, developing countries face complex circumstances such as resource constraints, policy differences, and cultural distinctiveness, which may result in intervention mechanisms and outcomes that are markedly different. As the largest developing country, China exhibits the distinctive characteristics of such nations. Therefore, examining the impact of early educational interventions on cognitive abilities within the Chinese context holds significant empirical value for enriching early education theory.

China’s educational methods and curriculum differ significantly from those of other countries. Unlike the neoliberal educational models prevalent in Western nations (Angus, 2011), China has long been dominated by an exam-oriented education system (Li K. et al., 2022; Li X. et al., 2022). As the largest developing country, China’s early educational sector faces constraints compared to developed nations, including limited resources, insufficient policy support, difficulties in meeting demand in terms of coverage and quality, and a lack of standardization in teacher training and curriculum (Khan et al., 2019; Krumins and Cakula, 2020). It also exhibits unique characteristics related to the household registration system, curriculum design, and exam-oriented education (Guo, 2020; Dai, 2022). At the same time, factors such as teacher-child interactions in early education interventions, family background, urban–rural disparities, teaching quality, and social–emotional development collectively influence the effectiveness of interventions (Hu et al., 2017; Yan et al., 2021; Qian, 2013; Zhai and Gao, 2008; Chen and Li, 2024). Among these, China’s unique household registration system has a profound impact on early educational interventions. The household registration system is a key mechanism for population management and the distribution of social welfare in China, defining individuals’ rights to engage in social and economic activities in specific locations. It is divided into urban, rural, and unified resident household registrations. Residents with rural household registration face restrictions on accessing public services in urban areas, whereas those with urban household registration can access benefits such as high-quality education, healthcare, and housing. The unified resident household registration is a product of China’s household registration reform, aims to achieve equal access to public services (Chan and Zhang, 1999). However, children from rural and unified resident households often remain unable to enroll in public schools due to cost barriers (Chen and Feng, 2013). At the same time, the high fees of formal private educational institutions force many children to attend unlicensed private schools, resulting in weaker cognitive abilities that required early intervention to improve (Wang et al., 2024). In addition, Chinese characters are characterized by their strong ideographic nature, flexible grammar, and rich semantics (Zhuang et al., 2018; Ma et al., 2020). The learning process requires memorizing a large number of vocabulary items and structures, which helps develop memory and comprehension skills (Fisher and Frey, 2014; Xiao and Ho, 2020). Unlike the English alphabet, which is easy to start with but becomes progressively more difficult (Chen et al., 2019), Chinese characters are challenging to learn initially, but can be memorized intuitively as learning progresses. Currently, most research on early educational interventions is based on developed-country contexts, and the mechanisms and effects of such interventions in China still require further investigation.

This study focuses on the mechanisms of early educational interventions on children’s cognitive development in China. The data on children’s reading and family activities provided by Peking University’s Open Data Platform covers many aspects of early educational interventions and children’s cognitive abilities. The data enables the study to control the important factors mentioned in the previous literature, and then analyzes the mechanism of early educational intervention in depth. The study aims to reveal the long-term effects of early educational interventions before age three on children’s cognitive abilities within a specific socio-cultural context in China. It also examines the heterogeneity effect among China’s household registration system and household income levels. Furthermore, the study explores the multiple ways in which early educational interventions affect children’s cognitive abilities through mediating mechanisms.

The marginal contributions of this study are mainly in three aspects. First, based on Chinese data, this study provides an in-depth analysis of the impact of early educational interventions before age three within China’s unique socio-cultural context. While numerous studies have explored the impact of early educational interventions on children’s cognitive ability, most have focused on developed countries such as Europe and the United States. There are few relevant empirical studies on China. Second, based on China’s household registration system, this study deeply discusses the heterogeneous effects of early educational interventions across different households. Third, the study delves into the heterogeneous effects of early educational interventions on different annual household income groups based on the income class divisions published in the National Economic and Social Development Statistics Bulletin issued by the National Bureau of Statistics of China. Fourth. this study comprehensively analyzes the mechanism of early educational intervention’s effects on children’s cognitive development through two mediating variables: children’s ability to read (R), and write Chinese characters (WW) before school enrollment, which enriches the empirical research in this field.

## 2 Theoretical mechanisms and research hypotheses

### 2.1 Early educational interventions and children’s cognitive development

Extensive research indicates that children’s brains undergo rapid neural development during the first 3 years after birth, a period referred to as the “sensitive period” of brain development (Perumal et al., 2021). During this period, the brain exhibits high plasticity, making environmental input particularly influential. Accordingly, early educational interventions play a crucial role in cultivating children’s cognitive abilities (Birbili, 2013). Such interventions not only enhance children’s cognitive development but also improve their problem-solving, logical reasoning, and language skills (Wang and Zheng, 2022; Muela et al., 2017). However, existing research suggests that traditional classroom instruction alone may be insufficient to stimulate complex cognitive development in early childhood (Pîrciu and Nitulescu, 2025). At the same time, the effectiveness of such interventions depends on long-time commitment. The benefits of short-term interventions are relatively limited and prone to fading, as children lack sufficient time to internalize and apply what they have learned (Resa et al., 2016; Hayes et al., 2013). Furthermore, Goldman et al. (2006) demonstrated that interventions from ages 0 to 3 had minimal long-term effects on cognitive, educational, and behavioral outcomes at ages 8 and 18. Therefore, it is reasonable to infer that early childhood interventions before age three follow a U-shaped relationship with the development of children’s cognitive abilities.

*H1*: There is a U-shaped relationship between early educational interventions before age three and children’s cognitive abilities.

### 2.2 Early educational interventions and the Chinese household registration system

China’s household registration system significantly impacts the equity of educational opportunities, which results in a systematic stratification of access to early educational interventions between urban and rural families. Urban families typically enjoy higher densities of quality early educational intervention institutions, specialized teachers, and standardized curricula (Magnuson and Shager, 2010). This allowed early educational interventions in urban families to promote cognitive development through high-quality learning environments. In contrast, rural and unified resident households faced challenges in providing early educational interventions due to factors such as low population density and high seasonal volatility (Vilches, 2023). Moreover, policy-driven urbanization strategy and chronic underinvestment in rural education exacerbated the structural divide between urban and rural children in terms of access to schooling, educational resources supply, and human capital transformation efficiency (Connelly and Zheng, 2007). In addition, financial constraints in rural households significantly hinder their capacity to afford early educational interventions. Furthermore, the household registration system also affects family educational concepts and practices. Families with urban household registration generally place greater trust in early educational interventions and are more willing to invest time and resources to promote children’s cognitive development. Therefore, there are some heterogeneous effects of early educational interventions on the Chinese household registration system.

*H2*: Early educational intervention has a heterogeneous effect on students with different family households.

### 2.3 Early education interventions and annual per capita household income

When exploring the impact of early education interventions on children’s development, the annual per capita household income may significantly affect the quality and effectiveness of the interventions received by children. In China, the distribution of educational resources is significantly influenced by family economic status (Abuzaid et al., 2022), which not only affects the equity of educational opportunities but also directly influences the quality of children’s early development. Different levels of annual per capita household income reflect differences in their ability to access educational resources. Higher-income families are usually able to provide richer educational resources and better quality educational environments, thus promoting children’s cognitive, linguistic, and social development (Decker et al., 2020). Children in these families are often exposed to more learning opportunities, such as private early childhood programs, rich reading materials, and professional educational instruction, which contribute to their early competencies. In contrast, low-income families may have difficulties in providing the same level of educational resources for their children due to financial constraints. This lack of resources may limit the development of children’s early competence and affect their cognitive and social skills. However, appropriate early education interventions may have a catalytic effect on these children, helping them bridge developmental gaps resulting from their families’ economic conditions (Wallander et al., 2014). The picture is more complex for middle-income families. While these families may not be able to devote as many resources to early learning as higher-income families, they are usually able to provide basic educational resources. However, due to limited resources, children in these families may not have access to the same level of early educational interventions as children in high-income families, potentially leaving some gaps in their early development. Thus, the impact of early learning interventions on the cognitive abilities of children in middle-income families may be less significant. Thus, there is some heterogeneity in the effect of early education intervention on annual income per capita.

*H3*: There is a heterogeneous effect of early education intervention on students from different family incomes.

### 2.4 Mediating mechanisms for early educational interventions

Existing studies have shown that brain plasticity is extremely high during the sensitive period of children’s development (0–3 years old) (Oh et al., 2022). The development of language and cognitive ability in this period has a profound impact on subsequent academic achievement and cognitive function.

In the Chinese context, early educational interventions during this sensitive period are particularly important. The complexity of the Chinese language demanded greater flexibility and adaptability in children’s linguistic and cognitive abilities (Chien et al., 2003). Its unique phonological, semantic, and written structures as an ideographic writing system place higher demands on children’s language learning (Su et al., 2018). Therefore, the role of promoting children’s cognitive development by interfering with reading ability (R) and writing Chinese characters ability (WW) has been widely confirmed. Among them, reading ability (R) is determined by the average value of children’s Pinyin recognition ability (PY), word reading Chinese characters ability (RW), sentence reading ability (RS) and Reading story skills (RG). The existing literature showed that: Training children’s Pinyin Recognition Skills (PY) can promote the development of memory and comprehension (Li K. et al., 2022; Li X. et al., 2022; Cheung et al., 2001). It can also enhance children’s sensitivity to phonology and help them to better grasp the rules for pronouncing Chinese characters during the sensitive period, which in turn promoted the overall development of language skills. Training children’s reading Chinese characters ability (RW) can exercise visual perception, which improved word extraction rate and attention (Li K. et al., 2022; Li X. et al., 2022). Meanwhile, training children to develop sentence-reading skills (RS) and stories-reading skills (RG) enhanced contextual reasoning (Bornkessel-Schlesewsky and Schlesewsky, 2013), improved sentence-reading skills and stories-reading skills enable children to better understand sentence structure and semantic relationships, thus facilitating effective contextual reasoning during the reading process. Additionally, training children to write Chinese characters (WW) benefited letter recognition, and consolidated children’s executive and cerebral responses. Moreover, handwriting may contribute to young children’s reading acquisition, which enhanced children’s cognitive abilities (Packard et al., 2006). The cascading optimization of these skills constitutes a mediating pathway of cognitive development that implicitly influences children’s cognitive improvement.

*H4*: Reading ability (R) and writing Chinese characters ability (WW) have mediating effects between early educational interventions and children’s cognitive ability.

*H4(a)*: Cultivating children’s reading ability can further improve cognitive ability by promoting their understanding and contextual reasoning ability.

*H4(b)*: Developing children’s Chinese character writing skills further enhances their cognitive abilities by promoting their executive skills.

## 3 Materials and methods

### 3.1 Procedures and participants

The data is sourced from the Reading and Elementary School Student Development Program on Peking University’s open data platform (Source: https://doi.org/10.18170/DVN/NNYSGT). The dataset was launched in December 2020 in all 36 elementary schools in District A of Hefei City, Anhui Province, sampling students in grades 4–6. Three classes in each grade were randomly selected (or all if insufficient) for the field study. In total, information on 13,069 students in 300 classes was collected. The group also selected the three counties in March 2021 that were the largest sources of migrant children in the urban sample. In each county, all townships were divided into three equal parts according to their economic level, and one township was randomly selected in each part. The sampling and research process for 4th-6th-grade students in the township elementary school was the same as that of the urban elementary school. A total of 2,586 students in 58 classes in nine elementary schools were collected. To ensure data quality, a data cleaning procedure was conducted to identify and remove responses with incomplete information or clear logical inconsistencies. Specifically, samples were excluded if respondents reported no preschool education while indicating a non-zero duration of attendance, or if elementary school students reported an age below 3 years. After applying these data validation criteria, 9,848 valid questionnaires were identified that met the requirements for analysis. Each sample’s questionnaires are categorized into student and parent questionnaires. The questionnaire source of each variable is shown in Table 1. The student participants in this survey range in age from with a mean age of 11.4 years, with 4,513 girls and 5,335 boys. There are 3,318 fourth graders, 3,288 fifth graders, and 3,242 sixth graders. There are 3,560 urban households, 4,193 rural households, and 2,095 unified residents.

**Table 1:** Definition of variables and descriptive statistics.

Variable name	Variable label	Mean	Std. dev.	Min	Max	Obs.	Questionnaire source
Language achievement (Y)	What was the student’s language grade last semester? A = 5; B = 4; C = 3; D = 2; E = 1	4.424	0.741	1	5	9,848	Student questionnaire
Math achievement (M)	What was the student’s math grade last semester? A = 5; B = 4; C = 3; D = 2; E = 1	4.315	0.996	1	5	9,848	
Cognitive ability (Cog)	(Y + M)/2	4.430	0.713	1	5	9,848	
Age	What is the age of the student?	13.371	1.009	9	18	9,848	
Grade	What is the student’s current grade? Grade 4 of primary school = 4; Grade 5 = 5; Grade 6 = 6	4.992	0.816	4	6	9,848	
Gender	What is the student’s gender? Man = 0; Woman = 1	0.458	0.498	0	1	9,848	
H	What is the student’s household type? Urban = 1; Rural = 2; Unified Resident = 3	1.851	0.743	1	3	9,848	
Participation in early education interventions (P)	Did the student go to an early childhood education center before age 3? Yes = 1; No = 0	0.180	0.384	0	1	9,848	Parent questionnaire
Duration of early education intervention (D)	How long has the student been enrolled in early learning and preschool programs? No participation = 1; up to 1 year = 2; 1 year = 3; 2 years = 4; 3 years = 5; 4 years and over = 6	3.484	1.873	1	6	9,848	
Educational expectations (PE)	What level of education do you want your child to achieve? Elementary school = 1; Junior high school = 2; Secondary school = 3 General high school = 4; College = 5; Bachelor’s degree = 6; Graduate school and above = 7	6.549	0.707	1	7	9,848	
Education level (PD)	What is your education level? Elementary school = 1; Junior high school = 2; Secondary school = 3 General high school = 4; College = 5; Bachelor’s degree = 6; Graduate school and above = 7	4.771	1.786	1	7	9,848	
Highest family education (HD)	What is the highest level of education in your family? Elementary school = 1; Junior high school = 2; Secondary school = 3 General high school = 4; College = 5; Bachelor’s degree = 6; Graduate school and above = 7	5.367	1.725	1	7	9,848	
Parent–child reading frequency (RF)	How often did you engage in parent–child reading with this student before he entered elementary school? Often = 1; sometimes = 2; never or hardly ever = 3	2.439	0.558	1	3	9,848	
Number of children’s books (BQ)	How many books for children are in your home? 10 or less = 1; 11–50 = 2; 51–100 = 3; 101–150 = 4; 151–200 = 5; 201–300 = 6; 301–400 = 7; 400 or more = 8	3.092	1.700	1	8	9,848	
Frequency of reading books (CF)	How often did children read books of his or her choice? Every day or almost every day = 1; Once or twice a week = 2; Once or twice a month = 3; Never or almost never = 4	3.637	0.664	1	4	9,848	
Read stories (RG)	How well did the student read stories when he or she entered elementary school? Very good = 4; Moderate = 3; Not very good = 2; Will not = 1	3.068	0.839	1	4	9,848	
Recognize pinyin (PY)	How well did children recognize pinyin when he or she entered elementary school? Very good = 4; Moderate = 3; Not very good = 2; Will not = 1	3.256	0.756	1	4	9,848	
Recognize Chinese characters (RW)	How well did children recognize Chinese characters at elementary school entry? Very good = 4; Moderate = 3; Not very good = 2; Will not = 1	3.104	0.834	1	4	9,848	
Identify sentences (RS)	How well did children identify sentences when he or she entered elementary school? Very good = 4; Moderate = 3; Not very good = 2; Will not = 1	3.174	0.830	1	4	9,848	
Write Chinese characters (WW)	How well did children write Chinese characters when he or she entered elementary school? Very good = 4; Moderate = 3; Not very good = 2; Will not = 1	3.023	0.848	1	4	9,848	
Read ability (R)	(PY + RW + RS + RG)/4	3.172	0.715	1	4	9,848	

Before completing the questionnaire, all participants were explicitly informed that their participation was based solely voluntary principle and that they were required to complete the relevant tasks independently in order to ensure the authenticity and objectivity of the data. All participants’ responses were kept strictly confidential and anonymous to protect their privacy. Written informed consent was obtained from all participants, and parents of study participants under 18 years of age provided informed consent.

### 3.2 Model construction

To explore in depth the effect of whether or not to choose an early education center for early educational interventions before the age of three on children’s cognitive ability, the equation 1 of this study is constructed as follows:


$$C_{\mathrm{og}}=\alpha +\beta_{1}X_{1}+\beta_{2}X_{1}^{2}+\beta_{3}X_{2}+\upsilon$$ (1)

Among them, α is a constant term, $\beta_{1}$ is the parameter to be estimated for whether or not a child chooses an early learning center for early educational interventions by age three, $\beta_{2}$ is a quadratic parameter to be estimated for whether to choose an early education center for early educational interventions before the age of three. $\beta_{3}$ is the parameter to be estimated for the control variable, and v is a disturbance term.

### 3.3 Measures

#### 3.3.1 Explanatory and explained variables

The explanatory variable is the interaction term ( $X_{1}$ ), defined as the product of Participation in early education interventions (P) and Duration of early education intervention (D). Cognitive ability ( $C_{\mathrm{og}}$ ) is used as an explained variable. Specifically, data are collected through a questionnaire, in which the questionnaire first asks, “Do the students attend an early education center before the age of three?” with answers of ‘Yes’ or ‘No’, intended to provide a preliminary understanding of the students’ acceptance of early education interventions. Subsequently, to further refine the level of participation in early education interventions, a second question is asked “What is the length of time the student has participated in early educational interventions? The answer options are “no participation,” “less than 1 year,” “1 year,” “2 years,” “3 years,” “4 years and above”. Based on the results of these two questions, we construct the interaction term $X_{1}$ to examine the moderating effect of intervention duration on the impact of early education participation. The construction of this interaction term helps us to analyze participation in early education interventions in depth and thus provides us with a more comprehensive and precise perspective for evaluating the effectiveness of early education interventions.

In the process of learning Chinese, children must memorize and comprehend a vast number of vocabulary words, sentence structures, and textual meanings, which helps develop their memory and comprehension skills (Fisher and Frey, 2014; Xiao and Ho, 2020). Language performance can reflect the strength of children’s cognitive abilities, such as memory and comprehension. At the same time, mathematics performance is highly correlated with logical reasoning, memory, and executive function (Geary, 2011; Bull et al., 2008; Nemmi et al., 2016). Since language development, logical reasoning, and executive function are core components of cognitive ability, this study uses language and mathematics scores as proxy variables for children’s cognitive ability. In assessing cognitive ability, we refer to the existing literature and find that previous studies used arithmetic averaging to comprehensively assess cognitive ability (Nemmi et al., 2016; Smith-Woolley et al., 2019). Based on this, the arithmetic mean of children’s language achievement (Y) and math achievement (M) is selected in this study as a composite measure of their cognitive ability ( $C_{\mathrm{og}}$ ).

#### 3.3.2 Control variables

This study introduces control variables ( $X_{2}$ ) that affect children’s cognitive abilities based on existing literature (Barbuscia and Mills, 2017; Gottfried, 2013; Nemmi et al., 2016; Johnson, 2013). The extrinsic variables include parental education (PD), parental expectation (PE), highest family education (HD), number of children’s books in the home (BQ), and parent–child reading frequency (RF). The intrinsic variables included the frequency of reading self-selected books (CF). Table 1 describes these variables. By scientifically controlling the relevant variables, we can more precisely assess the independent effects of early educational interventions before age three on children’s cognitive abilities.

#### 3.3.3 Mediating variables

In this study, children’s pre-school reading ability (R) and children’s ability to write Chinese characters before school (WW) are used as mediating variables. Among them, children’s pre-school reading ability (R) is the arithmetic average of the ability to recognize pinyin (PY), the ability to read Chinese characters before school (RW), the ability to read sentences before school (RS) and the ability to read stories before school (RG). Specific as shown in Table 1. The mediating variables are analyzed to determine their effects on children’s cognitive abilities. Specifically, we explore whether these variables mediate the impact of early educational interventions before age three on children’s cognitive abilities.

### 3.4 Statistical analysis

Stata/MP 14.0 is used for data preparation, management, and analysis. First, descriptive statistics are used to describe all variables and samples. Second, we conduct baseline regression analysis to examine the relationship between early education interventions and children’s cognitive abilities. The results show a U-shaped relationship with a significant positive effect (*p* < 0.01), it also verifies the H1. To test for heteroscedasticity, White’s test is used. The results show heteroskedasticity, so we use a robust standard error correction. The U-test shows that the core variables have negative primary terms and positive secondary terms, and the significance and goodness of fit were stable, with the “U-shaped” relationship robust. In addition, we conduct robustness tests by both shrinking and truncating the data by 1% (*p* < 0.01) and by adding control variables (*p* < 0.05). Immediately thereafter, we conduct a heterogeneity analysis to explore the heterogeneous effects of the Chinese household registration system and annual per capita household income on children’s cognitive abilities, and this part validates H2 and H3. Finally, we use a three-step approach to explore the mediating effect, and this part validates H4.

## 4 Results

### 4.1 Descriptive statistical analysis

The descriptive statistical analysis of this study covers the participation of 9,848 children in early educational interventions and their family background characteristics. According to the statistical results, as shown in Table 1. The participation rate of early educational interventions is low, and more than 80% of children do not receive early educational interventions before the age of three. This is similar to the situation in China. The widespread adoption of early educational interventions may be limited by high economic costs (Wong et al., 2013). The mean value of educational expectation (PE) is 6.549, reflecting that most parents generally have high expectations for their children’s educational level, and they tend to hope that children will obtain a master’s degree or higher in the future. And most of the families have university and higher degrees, which may have a positive impact on children’s educational environment. Meanwhile, more than 50% of the families have a high frequency of parent–child reading, which may contribute to children’s early cognitive development. In addition, Table 1 provides detail statistical characteristics of each of the children’s individual abilities, such as children’s cognitive abilities ( $C_{\mathrm{og}}$ ), children’s ability to recognize pinyin (PY) before school entry, children’s ability to read Chinese characters before school entry (RW), children’s ability to read sentences before school entry (RS), and children’s ability to write Chinese characters before school entry (WW). The index of cognitive ability is composed of the average value of Chinese achievement (Y) and mathematics achievement (M). It is worth noting that the scores are set by A-E in a context of China’s “double-reduction” policy, based on students’ raw scores, which are ranked and then divided into different grades, rather than directly publicizing students’ raw scores. This policy aims to effectively reduce the pressure and excessive academic burden caused by examinations. These statistics provide an important foundation for understanding the relationship between early educational interventions and children’s cognitive development.

### 4.2 Analysis of baseline regression results

In order to systematically examine the mechanism of the impact of early educational interventions before the age of three on children’s cognitive abilities, this paper adopts a stepwise regression method to regress the collected data. The models sequentially introduce control variables such as family background, reading environment, and children’s own abilities. Table 2 shows the regression results of the six nested models. Models 1 to 6 progressively incorporate the explanatory variables, Education level (PD) and Highest family education (HD), Educational expectations (PE), Parent–child reading frequency (RF) and Number of children’s books (BQ), Frequency of reading books (CF) and other control variables are gradually incorporated to examine their potential impact on the relationship between the explanatory variables and the explained variables.

**Table 2:** Linear regression model.

Variable	(1)	(2)	(3)	(4)	(5)
	cog1	cog1	cog1	cog1	cog1
X_1_	−0.030	−0.071^***^	−0.066^***^	−0.062^***^	−0.058^***^
	(−1.700)	(−4.271)	(−4.028)	(−3.847)	(−3.619)
X_1_^2^	0.007^*^	0.011^***^	0.010^**^	0.008^**^	0.008^*^
	(2.021)	(3.480)	(3.213)	(2.611)	(2.448)
PD		0.054^***^	0.042^***^	0.029^***^	0.028^***^
		(8.712)	(6.836)	(4.784)	(4.614)
HD		0.072^***^	0.060^***^	0.046^***^	0.044^***^
		(11.128)	(9.354)	(7.188)	(7.016)
PE			0.210^***^	0.186^***^	0.177^***^
			(21.196)	(18.806)	(18.105)
RF				0.074^***^	0.064^***^
				(5.876)	(5.148)
BQ				0.061^***^	0.054^***^
				(13.839)	(12.219)
CF					0.162^***^
					(16.085)
_cons	4.430^***^	3.797^***^	2.548^***^	2.476^***^	2.009^***^
	(559.165)	(167.964)	(40.473)	(37.609)	(28.218)
*N*	9,848	9,848	9,848	9,848	9,848
*r* ^2^	0.001	0.085	0.125	0.149	0.171
*F*	2.503	229.227	281.591	245.963	253.197

***p < 0.01, **p < 0.05, *p < 0.10.

Table 2 reports the baseline linear regression results. The results indicate a significant U-shaped relationship between the interaction term and the explained variable. Specifically, the coefficient on the linear term of the interaction is −0.058, which is negative at the 1% significance level, while the coefficient on the squared term is 0.008, which is positive at the 10% significance level. As the duration of early educational intervention increases, children’s cognitive abilities exhibit a pattern of “initial inhibition followed by promotion.” The model has a high overall fit, with an *R*^2^ of 0.171, and the F-statistic is significant, indicating that the model specification is reasonable. These results preliminarily support the research hypothesis. Regarding the regression results for the control variables, all coefficients are positive and significant at the 1% level, consistent with theoretical expectations. Among them, the coefficients for PD and HD are significantly positive, indicating that family educational background promotes cognitive development. Additionally, the coefficients for PE, RF, BQ and CF are also significantly positive, validating the positive effects of the family reading environment, parent–child interaction, and educational expectations, and further confirming the reasonableness of the model specification.

To better understand the practical implications of the U-shaped nonlinear relationship between early educational interventions before age three and children’s cognitive abilities, we calculate the turning point of the curve. Based on the results of the full-model estimation, this study estimates that the inflection point of the U-shaped relationship occurs at 1.625 years of early educational intervention. This value lies within the sample range, suggesting the existence of a threshold effect. Early educational interventions begin to have a positive impact on children’s cognitive abilities when they last for 1.625 years. When the duration is insufficient or the frequency too low, sporadic interventions struggle to produce a cumulative cognitive effect (Barnett, 1995). They may increase cognitive load due to fragmented learning, resulting in “ineffective education” or even “negative intervention” (Ramey and Ramey, 1998). From a practical perspective, the early education programs in China are typically organized in short-term, session-based formats (2–3 times per week). Sustained interventions are critical to achieve substantial effects. This study recommends that the cumulative duration of early educational interventions for children aged 0–3 years should not be less than 1.625 years, achieving cumulative cognitive stimulation through regular frequency. This approach avoids the “U-shaped trough” phenomenon, where intervention effects lag due to insufficient duration or premature discontinuation. In summary, there is a positive correlation between early educational interventions before age three and improvements in children’s cognitive abilities. H1 is valid.

The statistical evidence for the U-shaped relationship is subject to several limitations. Notably, the upward segment of the U-shaped relationship is statistically significant only at the 10% level. The descriptive statistics show that the mean duration of early educational interventions in the sample is 1.484 years, which is lower than the inflection point of 1.625 years. This suggests that a large proportion of samples are concentrated on the descending segment of the curve. This distribution pattern may limit the precision of estimates at the right tail of the curve. Therefore, the current findings provide only exploratory support for the U-shaped relationship, and the positive effects of long-term interventions require validation with larger samples (Table 3).

**Table 3:** Heteroscedasticity correction results for cognitive ability.

Variable	(1)	(2)
	Before correction	After correction
X_1_	−0.057^***^	−0.058^***^
	(−3.609)	(−3.679)
X_1_^2^	0.008^*^	0.008^*^
	(2.445)	(2.547)

Control variable	Control	
_cons	1.968^***^	2.009^***^
	(25.146)	(21.776)
*N*	9,848	9,848
*r* ^2^	0.171	0.171
*F*	225.258	203.749

***p < 0.01, **p < 0.05, *p < 0.10.

### 4.3 Heteroscedasticity test

To ensure the validity and accuracy of the regression results, this study conducts a heteroscedasticity test. Upon performing the White test, a *p*-value of 9.0e-81 is obtained, which is substantially smaller than the 0.01 significance level. Consequently, this study strongly rejects the null hypothesis that heteroscedasticity does not exist, indicating that the regression model exhibits significant heteroscedasticity. The presence of heteroscedasticity can lead to biased inference in OLS regression, including standard errors and *t*-values, potentially affecting the assessment of variable significance. In light of this, robust standard errors are employed to correct for this. Concurrently, a U-test is used to assess whether a U-shaped nonlinear relationship exists between the variables. The regression results before and after correction indicate that the coefficient for the linear term of the core explanatory variable is significantly negative. In contrast, the coefficient for the quadratic term is significantly positive. Furthermore, after correction using robust standard errors, the significance of the coefficients remains stable, and the goodness-of-fit remains unchanged. This demonstrates that the U-shaped relationship is robust. Further analysis of the U-test results reveals that while the slope at the upper bound of the interval is 0.034, with a *t*-value of 1.512 and a *p*-value of 0.065, it approaches significance at the 10% level. The overall test results support the existence of a U-shaped nonlinear relationship, indicating that early educational interventions before age three are associated with a U-shaped pattern in children’s cognitive abilities. This also supports hypothesis H1, demonstrating that early education interventions before the age of three are positively correlated with children’s cognitive abilities to some extent.

### 4.4 Robustness checks

In order to ensure the robustness of the findings, this study utilizes a combination of two methods for validation (see Table 4 for specific results), aiming to exclude potential errors and enhance the credibility of causal inferences. Firstly, the extreme values of the 1% before and after the sample are corrected by using the reduced-tail and truncated-tail treatment (Lin and Teng, 2023), which can reduce the interference of outliers on the regression results. Secondly, by adding additional control variables (Lin and Zhang, 2023), this study introduces potential confounders such as school library resources (SL) and teacher quality (ST). This method follows the principle of stepwise control and is able to systematically assess the risk of omitted variables. The results from both methods show that the coefficients of early educational interventions remain significantly positive, and the direction of the effect is consistent with the baseline model, confirming the robustness of the findings.

**Table 4:** Results of the U-shaped relationship test.

Variable	Lower bound	Upper bound
Interval	0	6
Slope	−0.058	0.034
*t*-value	−3.619	1.512
*P* > |*t*|	0.000	0.065

#### 4.4.1 Reduction and truncation of 1% before and after

In order to reduce the impact of outliers on the results of this paper, this paper chooses to shrink and truncate the samples before and after 1% to test the robustness of the findings. Specifically, shrinking the tails means replacing the values of the samples larger than the 99% quartile and the values of the samples smaller than the 1% quartile with the values of the samples at the 99% quartile and the values of the samples at the 1% quartile, respectively. Truncation is the process of replacing the values of the samples that are greater than the 99% quartile or less than the 1% quartile with the missing values and re-estimating the baseline regression using the shrunken and truncated samples. The regression results are shown in Model 1 and Model 2 of Table 5. From the results, it can be seen that the regression results in Model 1 and Model 2 do not change significantly from the benchmark regression results after the shrinking and truncation treatments, so the benchmark regression results are considered valid, and the empirical conclusions are robust.

**Table 5:** Robustness tests.

Variable	(1)	(2)	(3)
	1% before and after Y indentation	1% before and after Y truncation	Cog
X_1_	−0.0572^***^	−0.0555^***^	−0.0540^***^
	(0.0155)	(0.0143)	(0.0159)
X_1_^2^	0.0075^**^	0.0077^***^	0.0070^**^
	(0.0030)	(0.0028)	(0.0031)
PD	0.0283^***^	0.0271^***^	0.0256^***^
	(0.0059)	(0.0054)	(0.0060)
HD	0.0434^***^	0.0390^***^	0.0406^***^
	(0.0061)	(0.0057)	(0.0063)
PE	0.1703^***^	0.1432^***^	0.1731^***^
	(0.0095)	(0.0090)	(0.0098)
RF	0.0651^***^	0.0662^***^	0.0679^***^
	(0.0122)	(0.0113)	(0.0125)
BQ	0.0525^***^	0.0457^***^	0.0517^***^
	(0.0043)	(0.0039)	(0.0044)
CF	0.1570^***^	0.1316^***^	0.1584^***^
	(0.0098)	(0.0092)	(0.0100)
SL			0.0659^***^
			(0.0216)
ST			0.0007^***^
			(0.0001)
_cons	2.0756^***^	2.4243^***^	1.9221^***^
	(0.0695)	(0.0653)	(0.0751)
*N*	9848.0000	9699.0000	9848.0000
*r*^2^_a	0.1712	0.1565	0.1754

***p < 0.01, **p < 0.05, *p < 0.10.

#### 4.4.2 Method of adding additional control variables

In the process of conducting the robustness test, in order to further validate the reliability of the results of the baseline regression, this study adopts the method of adding additional control variables. In this paper, we introduce two new control variables into the original regression model: school library resources (SL) and school teacher quality (ST), and the results are shown in Model 3 of Table 5. The regression results remain consistent with the baseline regression results. Specifically, the regression coefficient for early educational interventions ( $X_{1}$ ) remains significantly negative, as does the coefficient for its quadratic term. This indicates that early educational interventions before age three exert a U-shaped effect on children’s cognitive abilities, consistent with the benchmark regression results. Additionally, the regression coefficients of the other control variables are also consistent with expectations. This further supports the existence of a positive relationship between family background and school environment on children’s cognitive ability to some extent. It also verifies that the benchmark regression results are valid and the empirical findings are robust.

### 4.5 Heterogeneity analysis

#### 4.5.1 Analysis of heterogeneity of family household registration

Considering the key role of the household registration system in China’s social structure, it not only affects an individual’s socioeconomic status but also profoundly shapes the access and quality of early education. In this study, the type of household registration is simplified to “rural” and “non-rural” because the urban–rural dichotomy remains an institutional watershed in the distribution of public services and resources, but the non-rural household registration in this study includes both the urban and the unified resident registration. This classification not only covers the traditional urban and rural households, but also includes the unified resident household, which emerged with the reform of the household registration system. These reflects the acceptance and effectiveness of early childhood education in different regions more comprehensively. The reason why differences in household registration type are chosen to explore differences in children’s cognitive abilities in this study is that defining the sample by school location alone will result in an urban group consisting of students from different household registries. Although these migrant children are currently enrolled in urban schools, their pre-migration experiences differ markedly from those of urban students (Xiong and Hu, 2022), resulting in differences in children’s cognitive abilities.

Table 6 shows the results. The findings indicate that in non-rural households, children’s early education interventions before age three have a significant positive impact on their cognitive abilities to some extent. Meanwhile, the regression coefficients of non-rural households on cultural capital indicators such as parent education, parent–child reading, and number of children’s books are all significant and generally higher than those in rural areas. This may be related to the abundance of early childhood education resources in urban areas, the quality of educational resources, and a general increase in the importance parents place on early childhood education interventions. Urban families tend to have access to more quality educational resources, and the quality of teaching and educational concepts in early childhood education centers is relatively mature, these factors work together to promote children’s cognitive abilities. At the same time, the cognitive abilities of children in unified resident households under early education interventions also show enhancement effects to a certain extent, and the household registration system makes it possible for children to have greater access to high-quality early education. This suggests that, with the reform of the household registration system, children with unified residential hukou have made some progress in accessing early education resources.

**Table 6:** Results of heterogeneity tests for family household registration.

Variable	(1)	(2)
	Rural Hukou	Non-rural Hukou
X_1_	−0.050	−0.058^***^
	(−1.408)	(−3.391)
X_1_^2^	0.001	0.009^**^
	(0.133)	(2.832)
PD	0.013	0.031^***^
	(1.152)	(4.114)
HD	0.037^***^	0.043^***^
	(3.658)	(5.330)
PE	0.177^***^	0.169^***^
	(11.471)	(13.232)
RF	0.061^**^	0.066^***^
	(2.748)	(4.429)
BQ	0.098^***^	0.036^***^
	(10.371)	(7.534)
CF	0.182^***^	0.146^***^
	(10.234)	(12.138)
_cons	1.907^***^	2.170^***^
	(16.127)	(23.638)
*N*	3,560	6,288
*r* ^2^	0.152	0.149
*F*	79.408	137.321

***p < 0.01, **p < 0.05, *p < 0.10.

In contrast, the cognitive ability of rural household families does not improve significantly, even if they receive early educational interventions before the age of three. This may be related to the lower investment in early educational resources and poorer quality of education in rural areas (Luo et al., 2009), as well as parents’ low awareness of early educational interventions. Early educational interventions in rural areas may not yet have formed an effective system, children have less access to educational resources, and the quality and content of teaching in early educational centers may not be able to meet the needs of children’s early development (Collins et al., 2016). Rural children face a double shortage of “low-quality early learning and low cultural capital”. The effects of the intervention cannot be sustained at home, which may lead to less effective early learning interventions. Consequently, less cognitive improvement in the children’s cognitive abilities.

The results of the comprehensive analysis show that children from different household backgrounds have significant heterogeneity in their cognitive development after receiving early educational interventions. Thus, the correctness of hypothesis H2 is verified.

#### 4.5.2 Heterogeneity analysis of annual per capita household income

To explore whether annual per capita household income has a heterogeneous effect on children’s cognitive ability, this study categorizes per capita income into three brackets based on the National Economic and Social Development Statistical Bulletin issued by the National Bureau of Statistics of China. These are the low-income group (annual per capita household income of $10,000–25,000), the middle-income group (annual per capita household income of $25,000–100,000), and the high-income group (annual per capita household income of $100,000 or more).

The regression results show a coefficient of −0.153 (*p* < 0.1) for X_1_ and 0.027 (*p* < 0.1) for X_1_^2^ in the low-income group, suggesting a trend toward a “U-shaped” relationship but with weak significance. This may be because children from the poorest households are benefiting from targeted interventions (Orr et al., 2021), leading to early educational interventions that stimulate nonlinear gains in children’s cognitive abilities. In the middle-income group, the X_1_ coefficient is −0.040, the X_1_^2^ coefficient is 0.003, indicating a non-significant “U-shaped” relationship, and the coefficient on family interaction (PD) is significantly positive (0.027^***^). This may be because families in this income group are in a transitional stage of balancing economic pressures with educational commitments. The impact on children’s cognition is more evident through direct interaction channels, such as parent–child companionship, than through the nonlinear effects of educational interventions. In the high-income group, the coefficient of the primary term is 0.057 (*p* < 0.05), and the coefficient of the secondary term is 0.008 (*p* < 0.1), which shows a significant “U-shaped” relationship. This may be because high-income families have sufficient economic strength to provide diversified, high-quality early educational interventions and learning environments, and are more likely to have access to high-quality educational resources (de la Fuente et al., 2019). This allows the “U-shaped” effect of educational interventions on children’s cognitive ability to be fully manifested.

The present study finds that neither parental education nor the child’s gender shows significant differences in the development of children’s cognitive abilities. This literature reports similar findings (Chen et al., 2021; Franse et al., 2021; Kersey et al., 2018), which are not specifically addressed in this paper. This finding contrasts with conventional wisdom, such as the idea that parental educational attainment has a key effect on children’s cognitive development (Cave et al., 2022), among others. Therefore, we hypothesize that factors such as children’s acquired level of personal effort may be more critical in shaping their cognitive development in the later stages. In this paper, children’s pre-school reading ability (R) and their ability to write words in school (WW) are selected to further explore the influence of later efforts on children’s cognitive development. These variables can reflect children’s learning outcomes and cognitive foundations prior to school entry and are important indicators of children’s early learning effort. Thus, the correctness of hypothesis H3 is verified.

### 4.6 Intermediation mechanism test

In order to investigate the potential influence mechanisms of early educational interventions before the age of three on children’s cognitive abilities, we select two variables reflecting children’s early educational efforts: reading skills (R) and writing Chinese characters skills (WW) before school entry. These variables serve as important indicators of children’s learning outcomes and cognitive foundations before school entry. This study chooses to employ a three-step approach to test for mediating effects (Pan et al., 2023). The first step of the three step mediation effect test model is shown in equation 2, and the second and third steps are shown in equations 3–4:


$$Y=\alpha +\beta_{1}X_{1}+\beta_{2}X_{2}+\upsilon$$ (2)

$$C_{n}=\alpha +\beta_{1}X_{1}+\beta_{2}X_{2}+\upsilon$$ (3)

$$Y_{n}=\alpha +\beta_{1}X_{1}+\beta_{2}X_{2}+\beta_{3}C_{n}+\upsilon$$ (4)

This study explores the developmental path of children’s pre-school cognitive ability through two mediating variables, each measured as shown in Table 1. Specifically, when *n* = 1, $C_{n}$ represents children’s pre-school reading ability (R), which reflects children’s ability to understand the structure and semantics of language and also lays the foundation for the development of spelling ability. When *n* = 2, $C_{n}$ represents children’s pre-school writing word ability (WW), which is not only an important reflection of language output ability but also a comprehensive result of cognitive development. These two mediating variables cascade to form a developmental path from basic cognitive skills to comprehensive cognitive abilities. In this study, the conduction mechanism will be tested based on the values and significance levels of coefficient $\beta_{1}$ in model (2) and coefficients $\beta_{1}$ and $\beta_{2}\;$ in model (3). The results are shown in Table 6.

The results are shown in Table 8. The results in Column 1 of Table 8 show that the coefficient for the primary term of the early educational intervention is −0.058 (*p* < 0.01), and the coefficient for the secondary term is 0.008 (*p* < 0.1). This clearly shows a “U-shaped” nonlinear relationship between the early educational intervention and children’s cognitive ability. Specifically, at low levels of intervention, there is a significant negative effect on children’s cognitive ability. As the level of intervention increases, this effect gradually changes from negative to positive. After crossing this inflection point, the facilitating effect of early educational intervention on children’s cognitive ability begins to emerge. This result reveals that the effect of early educational intervention on children’s cognitive ability is not a simple linear relationship. Instead, there are stage differences, which provide an important basis for an in-depth understanding of the mechanism of early educational intervention.

**Table 8:** Mediating effects of early educational interventions by age three, cognitive abilities, and $C_{n.}$

Variable	(1)	(2)	(3)	(4)	(5)
	Cog	R	Cog	WW	Cog
*R*			0.109^***^		
			(11.362)		
WW					0.066^***^
					(8.247)
X_1_	−0.058^***^	0.044^**^	−0.062^***^	0.057^**^	−0.061^***^
	(−3.614)	(2.651)	(−3.940)	(2.853)	(−3.862)
X_1_^2^	0.008^*^	−0.003	0.008^*^	−0.005	0.008^*^
	(2.445)	(−0.800)	(2.552)	(−1.209)	(2.553)
PD	0.028^***^	−0.060^***^	0.034^***^	−0.099^***^	0.034^***^
	(4.615)	(−9.573)	(5.715)	(−13.051)	(5.667)
HD	0.044^***^	0.000	0.044^***^	−0.007	0.044^***^
	(7.020)	(0.057)	(7.059)	(−0.912)	(7.119)
PE	0.177^***^	0.095^***^	0.167^***^	0.095^***^	0.171^***^
	(18.114)	(9.340)	(17.086)	(7.726)	(17.480)
RF	0.064^***^	0.342^***^	0.027^*^	0.292^***^	0.045^***^
	(5.124)	(26.303)	(2.073)	(18.641)	(3.530)
BQ	0.054^***^	0.017^***^	0.052^***^	−0.006	0.054^***^
	(12.218)	(3.632)	(11.873)	(−1.088)	(12.349)
CF	0.162^***^	0.044^***^	0.157^***^	0.026^*^	0.161^***^
	(16.149)	(4.193)	(15.760)	(2.035)	(16.032)
_cons	2.007^***^	1.766^***^	1.814^***^	2.099^***^	1.868^***^
	(28.186)	(23.783)	(24.939)	(23.450)	(25.626)
*N*	9,852	9,852	9,852	9,852	9,852
*r* ^2^	0.171	0.103	0.182	0.073	0.177
*F*	253.617	140.689	242.714	97.132	234.528

***p < 0.01, **p < 0.05, *p < 0.10.

#### 4.6.1 The mediating role of R on children’s cognitive abilities

The results in columns (2) and (3) of the table reveal the mediating mechanism of children’s pre-school reading ability (R) in the effect of early educational intervention on children’s cognitive ability. The analysis in Column (2) shows that the regression coefficient of the primary term of early learning intervention on children’s pre-school reading ability (R) is 0.044 (*p* < 0.05). It indicates that an increase in the duration of early educational intervention can significantly and positively promote the development of children’s pre-school reading ability. This indicates that adequate early educational intervention can effectively enhance children’s reading ability, particularly in language initiation and the development of reading habits, consistent with many studies (Lingwood et al., 2020; Sucena et al., 2023). Meanwhile, the coefficient for the quadratic term of early learning intervention is −0.003, not significant, indicating that the effect of early learning intervention on children’s pre-school reading ability (R) tends to be more positive and linear under the current modeling setting. The results in Column (3) show that the regression coefficient of children’s pre-school reading ability (R) on children’s cognitive ability (Cog) is 0.109 (*p* < 0.01), which suggests that there is a significant positive effect of children’s pre-school reading ability on cognitive ability. In terms of cognitive development, reading ability is an important vehicle for developing children’s cognitive capacities, such as information acquisition, logical thinking, and language comprehension (Helder et al., 2016; Hasan, 2023). Good reading ability can continuously promote children’s cognitive ability through pathways such as enriching knowledge reserves and exercising thinking skills (Kuntze et al., 2014). Combining the results in columns (2) and (3), children’s pre-school reading ability (R) plays a significant mediating role in the effect of early education hours on children’s cognitive ability. Therefore, early educational intervention can positively promote children’s cognitive development to some extent by enhancing their pre-school reading ability. Hypothesis H4(a) is verified (Table 7).

**Table 7:** Results of heterogeneity tests for annual per capita household income.

Variable	(1)	(2)	(3)
	10–25 thousand	25–100 thousand	More than 100 thousand
X_1_	−0.153^*^	−0.040	−0.057^**^
	(−2.539)	(−1.577)	(−2.837)
X_1_^2^	0.027^*^	0.003	0.008^*^
	(2.227)	(0.589)	(2.153)
PD	−0.001	0.027^**^	0.031^**^
	(−0.050)	(3.121)	(3.163)
HD	0.059^***^	0.043^***^	0.027^*^
	(3.441)	(4.982)	(2.401)
PE	0.187^***^	0.196^***^	0.109^***^
	(7.084)	(14.584)	(6.360)
RF	0.088^*^	0.035^*^	0.104^***^
	(2.359)	(1.975)	(5.446)
BQ	0.100^***^	0.058^***^	0.037^***^
	(5.903)	(8.690)	(6.451)
CF	0.150^***^	0.189^***^	0.113^***^
	(4.911)	(13.376)	(7.259)
_cons	1.827^***^	1.847^***^	2.720^***^
	(9.275)	(18.749)	(21.734)
*N*	1,222	5,256	3,370
*r* ^2^	0.171	0.167	0.113
*F*	31.294	131.490	53.302

***p < 0.01, **p < 0.05, *p < 0.10.

#### 4.6.2 The mediating role of WW on children’s cognitive abilities

The results in columns (4) and (5) of the table clearly present the mediating mechanism of children’s Chinese character writing skills (WW). The regression results in column (4) show that the coefficient of the primary term of early educational intervention on children’s pre-school writing ability (WW) is 0.057 (*p* < 0.05). This significant positive relationship indicates that children’s Chinese character writing skills can be effectively improved with the increase in the duration of early educational interventions, reflecting that adequate early educational intervention can support the development of children’s writing ability in terms of writing norms guidance. Moreover, the coefficient of the quadratic term is - 0.005, which does not pass the significance test, indicating that the main effect is a linear relationship. In column (5), the regression coefficient of children’s Chinese character writing skills (WW) on children’s cognitive ability (Cog) is 0.066 (*p* < 0.012), which indicates that children’s Chinese character writing skills have a significant positive contribution to their cognitive ability. From the perspective of cognitive development, writing ability involves the synergistic development of children’s multidimensional abilities such as hand-eye coordination, spatial cognition, and logical thinking. The development of children’s Chinese character writing skills indirectly affects cognitive ability by enhancing children’s executive ability, which involves planning, organizing, monitoring, and regulating abilities (Luchsinger et al., 2021). In summary, children’s Chinese character writing skills (WW) play a significant mediating role in the effect of early educational duration on children’s cognitive ability. Specifically, early education duration positively promotes children’s cognitive development by enhancing their pre-school writing ability, thereby supporting H4(b).

## 5 Discussion

Based on the tracking data of 9,848 children in Hefei City, we examine the effect of early educational intervention before the age of three on children’s cognitive ability and its mechanism of action are verified by constructing a binary equation. The results of the study show that early educational intervention before age three has a significant U-shaped relationship with children’s cognitive ability, and this relationship is heterogeneous across children with different household types and family incomes. In addition, this study explores the multiple ways in which early educational interventions affect children’s cognitive abilities through mediating mechanisms. Two mediating variables, children’s pre-school reading ability (R) and children’s Chinese character writing skills (WW), are found to mediate the effects between early educational interventions and children’s cognitive abilities (Table 8).

### 5.1 The relationship between early educational interventions before age three and children’s cognitive abilities

The results of this study indicate that there is a significant U-shaped relationship between early educational intervention before age three and children’s cognitive abilities. To some extent, it verifies that there is a positive relationship between early educational intervention before age three and children’s cognitive ability. H1 is confirmed. This result is similar to the findings of a previous study (Campbell et al., 2001; Barnett, 1998; Ma et al., 2024; Pietropoli and Gracia, 2025), which supports the idea that early educational interventions can positively predict children’s cognitive abilities. Early educational interventions can provide children with a rich learning environment. This environment, whether created through the optimization of the home environment, professional guidance from educational institutions, or the full use of community resources, enables them to achieve holistic development in areas such as logic, language, and memory (Passolunghi and Costa, 2016; Blair, 2016). Specifically, early educational interventions have a positive effect on improving children’s language achievement, which not only enhances their comprehension but also contributes to the overall improvement of cognitive skills (Bierman et al., 2008). Additionally, early educational interventions are associated with improved children’s math scores, which not only helps to enhance their logical reasoning and executive functioning, but also contributes to the development of cognitive abilities (Yamauchi and Liu, 2012). These findings emphasize the important role of early educational interventions in children’s cognitive development and provide a strong evidence base for future educational practice and policy development.

### 5.2 The role of heterogeneity in China’s household registration system

The study finds that there is a heterogeneous effect of the Chinese household registration system on the impact of early educational interventions before age three and children’s cognitive abilities, confirming H2. Based on the localized institutional context of China’s household registration system, this study distinguishes itself from previous literature that focuses only on the impact of rural versus urban household registration on children’s cognitive development. The household registration in this study covers the Unified Resident Hukou to investigate whether China’s household registration reform affects children’s cognitive ability. The results show that there are differences in the development of children’s cognitive abilities among families with different hukou. The effect of early educational intervention on children’s cognitive ability is more significant for children with urban or unified resident hukou, but not for those with rural hukou. This may be due to the uneven distribution of early educational resources, the varying quality of early educational services, and the differences in the interaction between the home environment and early educational services, which in turn affect the actual effects of early educational interventions. It is worth noting that the significance of the early education intervention effect of the unified resident hukou, as a product of China’s household registration reform, suggests that the reform promotes the optimal allocation of educational resources to some extent.

### 5.3 The role of heterogeneity in annual per capita household income

The study finds that annual per capita household income has heterogeneous effects on the association between early educational interventions and children’s cognitive abilities before age three, confirming H3. In this study, given the National Economic and Social Development Statistical Bulletin published by the National Bureau of Statistics of China, per capita income is categorized into low, medium, and high brackets. The results show differences in children’s cognitive development across families with different incomes. The study finds that children from high-income families show significant improvement in their cognitive abilities after receiving an early educational intervention, indicating that sufficient economic resources can provide adequate material and environmental support for the intervention and magnify its effects. Children from low-income families also show significant cognitive gains, which may be attributed to the intervention’s effective supplementation of scarce resources. However, the cognitive development of children from middle-income families does not show significant changes, suggesting that this group may be in the “intervention blind zone”, lacking the resource compensation needs of low-income families and the quality support conditions of high-income families. Thus, annual per capita family income not only determines the richness of the early childhood environment but also directly shapes the boundaries of the effectiveness of early educational interventions, highlighting the importance of formulating differentiated and precise early education policies.

### 5.4 The mediating role of R, WW

Our study further demonstrated that early educational interventions can enhance children’s cognitive abilities by improving preschool reading ability (R) and Chinese character writing skills (WW), with significant mediating effects, confirming H4. Specifically, the cultivation of reading ability (R) before school entry, as an important pathway for children’s knowledge acquisition, has far-reaching significance. By intervening in children’s reading ability through early educational interventions, children can achieve significant improvements in language comprehension, vocabulary acquisition, and text interpretation, laying a solid foundation for their learning after entering school. Meanwhile, writing Chinese character ability (WW), as a key skill for children’s language expression and externalization of thinking, also plays an important role in children’s cognitive development. After school, children need to write to consolidate what they have learned and to express their ideas and opinions. Early educational interventions can help children develop good habits and norms in writing posture, stroke order, Chinese character structure, and other aspects, thereby improving their writing speed and accuracy. This improved writing ability not only helps children better master the use of Chinese characters in language learning but also promotes clear, logical thinking.

Selecting reading ability (R) and writing Chinese characters skills (WW) as mediating variables has important theoretical and practical implications. On a theoretical level, this finding enriches our understanding of the intrinsic mechanisms by which early educational interventions affect children’s cognitive development. It reveals how children’s cognitive development is gradually influenced through the two key aspects of reading and writing, providing a new perspective and theoretical basis for subsequent educational research. On a practical level, the results of this study provide a clear direction for early educators and parents. By emphasizing the cultivation of children’s reading and writing Chinese character skills in early educational interventions, children’s cognitive abilities can be effectively enhanced, laying a solid foundation for their future learning and growth.

## 6 Conclusion

This study analyzes in depth the positive impact of early educational interventions on children’s cognitive abilities. Meanwhile, based on China’s household registration system and the National Economic and Social Development Statistics Bulletin published by the National Bureau of Statistics of China. The effects of early education interventions on cognitive ability among children with different household registration types and annual per capita household incomes are analyzed in depth. In addition, this study empirically finds that two mediating variables, children’s pre-school reading ability (R) and writing Chinese characters skills (WW), mediated the effects between early educational interventions and children’s cognitive abilities before age three, which lays the foundation for the design of targeted interventions to improve children’s cognitive ability developmental outcomes.

This study has certain limitations that need to be addressed in future research. Although the samples in this study are selected through random sampling and are large in size, the sample is limited to Hefei City, Anhui Province. This geographical limitation restricts the broad applicability of the findings to a certain extent, so that they may not fully reflect the actual situation in all regions of China. Therefore, subsequent studies should consider expanding the scope of the survey to other regions of the country to enhance the broad applicability and representativeness of the findings. Second, due to limitations in data resources, this study is unable to fully explore all possible mediating variables and confounding factors. Furthermore, against the backdrop of China’s “Double Reduction” policy, raw score data is difficult to obtain due to privacy concerns, making the A-E grading system the optimal feasible solution for this study in terms of data accessibility and policy alignment. However, the use of grade bands may result in loss of variance and may not be sufficient to capture more subtle differences. Future research may consider incorporating more diverse and extensive datasets or employing more advanced statistical analysis methods. This would help further validate the effects of early educational interventions before age three. Ultimately, these approaches could more accurately reveal the causal relationship between early educational interventions and children’s cognitive development.

## Data Availability

Publicly available datasets were analyzed in this study. This data can be found at: https://opendata.pku.edu.cn/.
